# Assessment of quality of life among elderly in urban and peri-urban areas, Yangon Region, Myanmar

**DOI:** 10.1371/journal.pone.0241211

**Published:** 2020-10-29

**Authors:** Poe Ei Zin, Yu Mon Saw, Thu Nandar Saw, Su Myat Cho, Su Su Hlaing, May Thet Nu Noe, Tetsuyoshi Kariya, Eiko Yamamoto, Kay Thi Lwin, Hla Hla Win, Nobuyuki Hamajima

**Affiliations:** 1 Department of Healthcare Administration, Nagoya University Graduate School of Medicine, Nagoya, Japan; 2 Department of Preventive and Social Medicine, University of Medicine 1, Yangon, The Republic of the Union of Myanmar; 3 Nagoya University Asian Satellite Campuses Institute, Nagoya, Japan; 4 Department of Community and Global Health, the University of Tokyo, Tokyo, Japan; 5 South District Yangon Regional Health Department, Yangon, Myanmar; 6 Kaya State, Public Health Department, Ministry of Health and Sports, Loikaw, Kayah State, The Republic of the Union of Myanmar; 7 University of Public Health, Yangon, The Republic of the Union of Myanmar; Iwate Medical University, JAPAN

## Abstract

**Background:**

In the context of an aging population, quality of life (QOL) is an important consideration for the well-being of the elderly. However, there is limited information on the QOL of the elderly in Myanmar. This study aimed to explore the risk factors for low QOL among the elderly in urban and peri-urban areas of the Yangon Region, Myanmar.

**Methods:**

A community-based, cross-sectional study was conducted among the elderly aged 60 years or older in two urban and two peri-urban townships in the Yangon Region from July to September 2019. A multi-stage sampling method was used to recruit study participants using a pre-tested questionnaire. A total of 616 (305 males and 311 females) elderly people were interviewed using a face-to-face interview technique. Multiple linear regression analysis was performed on the four domains (physical health, psychological health, social relationship, and environment) of QOL measured with the WHOQOL-BREF.

**Results:**

Income level and having intimate friends influenced the QOL scores of the elderly in all domains, while education level and marital status influenced psychological health, social relationship, and environment domains. Social interaction with neighbors increased the QOL scores for physical health, social relationship, and environment domains. Living in peri-urban areas was associated with lower QOL scores for physical health, psychological health, and environment, while participation in group activities increased QOL scores in these domains. Having comorbidities affected the QOL for psychological health and environment domains, while the frequency of going out affected physical health, and the frequency of religious performance affected social relationship.

**Conclusion:**

Residential location, education level, marital status, income, comorbidities, social interactions with neighbors and friends, participation in group activities, and frequencies of going out and religious activities should be considered in planning and implementing programs for the elderly in Myanmar. Peri-urban development, strengthening healthcare and social security systems, and encouraging social interaction and participation in group activities play critical roles in improving the QOL for elderly residing in Myanmar.

## Introduction

The population aging is a global trend. The global population aged 65 years or older has increased from 6% in 1990 to 9% in 2019, and it is estimated to be 16% in 2050 [1]. Some of the countries in East and Southeast Asia have a very high aging rate, and it is estimated that the elderly population in those countries will be around double to 22% by 2050 from 11% in 1990 [1]. In the Association of Southeast Asian Nations (ASEAN), Vietnam will become an aged society within 19 years (one third of the total population will become elderly in 2050), and Singapore and Thailand will become the same in 22 years [2]. Such rapid growth in the elderly population presents challenges for governments in providing quality healthcare and social security, and also greatly affects societies and caregivers in many aspects.

Now that aging population has a longer life span than ever before, it is important to help individuals grow old with a good quality of life (QOL). The World Health Organization (WHO) defines QOL as “an individual’s perception of their position in life in the context of culture and value systems in which they live and concerning their goals, expectations, standards, and concerns” [3]. QOL also is described as a concept concerning physical health, mental health, social relationships, and emotional well-being [4]. QOL for elderly people is a combination of life-course and immediate influences and is highly subjective [5]. Further, QOL for the elderly may differ from that of other age groups because many factors influence their QOL. Several studies from Southeast Asia have pointed out that older age, having low education level, having insufficient income, being unemployed, having a current illness, alcohol consumption, and inactive daily living activity were risk factors associated with lower QOL among the elderly [6–8]. Understanding the factors influencing QOL for the elderly population is important information for countries’ policy makers, planning, and implementation of healthcare and other supporting programs for the elderly.

In Myanmar, a developing country in Southeast Asia, the elderly population has been increasing and Myanmar government has been trying to improve its health and social security systems since 2015 to match its expanding elderly population. The proportion of elderly (60 years and older) in Myanmar was 6.0% in 1973, 6.4% in 1983, and 8.9% in 2014. Which is estimated to increase to 15.5% in 2035 and 20.2% in 2050 [9–12]. The life expectancy for both sexes in Myanmar was 66.8 years in 2018 [13]. A previous study in 2014 reported that older people in Myanmar were quite poor in terms of material well-being, lived in low-income households, and had poor or very poor self-reported health [14]. Financial hardship and poor health in the elderly of Myanmar could negatively impact overall QOL for the elderly.

Past studies on QOL for the elderly in one township from the Irrawaddy Division and one from the Bago Region in Myanmar reported that self-esteem, family relationships, and individual income were significantly associated with the elderly’s QOL [15, 16]. However, QOL and the associated factors may be different for elderly residing in big cities like Yangon, a city with largest elderly population and 14.4% of the elderly aged 60 years and over were residing [11, 12]. Forty-nine percent of the people who live in urban areas of Myanmar aged 60 years and over live in major cities such as Yangon and Mandalay [12]. However, there is limited information on QOL and its associated factors among the elderly population living in Myanmar’s metropolitan areas. In an effort to provide evidenced-based information for developing and implementing policies and plans for the growing elderly population, the current study aimed to examine the factors associated with QOL among the elderly in urban and peri-urban areas of the Yangon Region according to four domains of the World Health Organization’s Quality of Life short form (WHOQOL-BREF).

## Methods

### Study area and participants

A community-based, cross-sectional study was conducted in the Yangon Region of Myanmar from July to September 2019. The Yangon Region has 45 townships comprising 27 urban and 18 peri-urban townships. In accordance with the United Nations' standard for being “aged” in both developed and developing countries, and the official retirement age in Myanmar, this study defined elderly as aged 60 years and older [17]. The eligibility criteria for participants were as follows: 1) aged 60 years or older, 2) residing in the survey area at least six months before the survey, 3) having no severe cognitive impairment, and 4) being physically and mentally sound. A total of 672 elderly individuals were invited for interview. Of these, 640 gave consent to participate in the study; a response rate of 95.2%. After data cleaning, 24 participants were excluded from the dataset due to missing responses for the outcome variables. In total, 616 elderly participants (305 men and 311 women) were included in the final analysis.

### Data collection

This study used multi-stage sampling. Of the seven states, seven regions, and one territory in Myanmar, the Yangon Region was selected because it is the most populated with the largest elderly population; over 600,000 elderly population [18]. The Yangon Region is composed of four districts: east, west, south, and north [18]. The east and west districts are central and urbanized areas in the Yangon Region, while the south and north districts are peripherally located. In the second stage, four townships were randomly selected using a lottery method: Lanmadaw township from the east, Dawbon township from the west, and Dala and Twantay townships from the south. Third, three wards from each township were selected using a lottery method. Fourth, to obtain a sufficient sample size, 60 households were selected by house-to-house sampling after a random selection of a starting point. Finally, one eligible elderly participant from each selected household was randomly recruited. If there was no eligible person in the selected household, then the next household with an eligible elderly participant was chosen. Data were collected through face-to-face interviews using pre-tested semi-structured questionnaires. The questionnaires consisted of three parts: 1) background characteristics, 2) social characteristics, and 3) the World Health Organization’s Quality of Life short form (WHOQOL-BREF).

### Dependent variables

QOL was assessed using the 26-item WHOQOL-BREF, which contains overall QOL and general health (2 items), physical health (7 items), psychological health (6 items), social relationship (3 items), and environment (8 items) domains. Each item was evaluated using a five-point Likert scale. The scores for each domain was calculated by adding the mean score values for single items to be compatible with WHO’s QOL assessment (WHOQOL-100). Values were transformed into scores ranging from 0 to 100 according to the WHO guidelines. The questionnaires were pre-tested among 60 elderly individuals from the Yangon Region other than the selected townships. Cronbach’s alpha for the WHOQOL-BREF questionnaire after the pretest was 0.89. The pretest results were used to modify and adjust according to the local context and study population. After modification, the Cronbach’s alpha of the WHOQOL-BREF questionnaire was 0.91.

### Independent variables

In the current study, background and social characteristics were the independent variables. Background characteristics included age, gender, residence, religion, education, occupation, working status, income, marital status, family type, and comorbidities. Interaction with the neighborhood, having intimate friends, praying/worshiping/meditation, frequency of donation, frequency of going out, and participation in group activities were included as social characteristics.

Age was categorized into two groups: 60 to 69 years and 70 years or over. Religion was divided into Buddhist and others (Hindu, Christian, and Islam). The participants were divided into groups based on their marital status (single, married: meaning currently living with a spouse, and separated/widowed/divorced), occupation (private/public sector, self-employed, and never had a job), and family type (nuclear family, joint or extended family, and three-generation family). Using the median income (60,000 kyats), the participants were divided into a high-income group, a low-income group, and no income group. Regarding comorbid conditions, the participants were divided into four groups: those with no disease or no knowledge of having a current disease, those with one disease, those with two diseases, and those with three or more diseases.

Regarding praying/worshiping/meditation, the participants were divided into two groups: those performing these activities daily and those not performing them daily. The frequencies for donation and going out were categorized as donating or going out once a week or more and less than once a week.

### Statistical analysis

After data collection, most responses to the questionnaires were pre-coded. After checking the data, the coded data were entered into the computer using EpiData software version 3.1. Errors in data entry were checked by reviewing the questionnaires. Statistical analysis was performed using the Statistical Package for Social Sciences (SPSS) software program version 22.0 (IBM SPSS Inc.). Descriptive statistics and summary statistics were calculated. For categorical data, frequency and percentage were calculated. The mean and standard deviation (SD) were calculated for continuous data. An independent *t*-test was performed to compare the QOL scores between the two groups. Multiple linear regression analysis was performed using the enter method to determine the association between the independent variables and QOL. Coding for the qualitative independent variables, such as education, occupation, income, marital status, type of family, comorbidities, interaction with the neighborhood, and having intimate friends as either ^“^0^”^ or ^“^1^”^ as dummy variables. These coded qualitative variables were included as additional independent variables in the multiple linear regression analysis. Statistical significance was set at 0.05, and statistical tests were conducted to ensure that the necessary assumptions were met.

### Ethical considerations

Ethical approval was obtained from the Institutional Technical and Ethical Review Committee at the University of Public Health, Ministry of Health and Sports, Myanmar [Ethical No. UPH-IRB (2019/Research/32) issued July 30, 2019]. The survey was conducted according to committee guidelines. Before obtaining informed consent, it was ensured that participants understood the nature, purpose, and possible benefits of the study. Participants were also informed of the duration of the interview, the individual right to withdraw from the study at any time without penalty, and the confidential handling of the survey information. If there was an abnormal finding during the interview, the participants were counseled and appropriately advised of any necessary interventions.

## Results

Table 1 presents the background characteristics of the participants by gender. Overall, 305 participants (49.5%) were men, and 311 (50.5%) were women. Of these, 57.0% of the males and 57.2% of the females were aged 60–69 years. Nearly half of the participants (51.5% of the males and 53.1% of the females) lived in peri-urban areas of the Yangon Region. The majority of participants (95.1% of the males and 96.5% of the females) were Buddhists. Regarding education, 31.8% of the males reported high school level or above, while 40.8% of female participants had a primary school level education. The longest occupation was listed as self-employed for 59.0% of male participants and 41.8% of female participants. Half of the participants (54.8% of the males and 51.4% of the females) lived in a nuclear family. Regarding comorbidities, 44.9% of the males and 35.7% of the females reported that they had at least one disease.

**Table 1:** Background characteristics of the study participants.

Characteristics	Total		Male		Female	
	(N = 616)		(N = 305)		(N = 311)	
	N	(%)	n	(%)	n	(%)
**Age (years)**						
60–69	352	(57.1)	174	(57.0)	178	(57.2)
≥70	264	(42.9)	131	(43.0)	133	(42.8)
**Residence**						
Urban	294	(47.7)	148	(48.5)	146	(46.9)
Peri-urban	322	(52.3)	157	(51.5)	165	(53.1)
**Religion**						
Buddhist	590	(95.8)	290	(95.1)	300	(96.5)
Others	26	(4.2)	15	(4.9)	11	(3.5)
**Education**						
No school/only read and write	140	(22.7)	63	(20.7)	77	(24.8)
Primary school level	203	(33.0)	76	(24.9)	127	(40.8)
Middle school level	115	(18.7)	69	(22.6)	46	(14.8)
High school level and above	158	(25.6)	97	(31.8)	61	(19.6)
**Type of occupation**						
Private/public sector	245	(39.8)	123	(40.3)	122	(39.2)
Self-employed	310	(50.3)	180	(59.0)	130	(41.8)
Never had a job	61	(9.9)	2	(0.7)	59	(19.0)
**Working status**						
Currently working	135	(21.9)	77	(25.2)	58	(18.6)
Not currently working	481	(78.1)	232	(74.8)	256	(81.4)
**Income**						
No income	258	(41.9)	119	(39.0)	139	(44.7)
Low income	259	(42.0)	138	(45.2)	121	(38.9)
High income	99	(16.1)	48	(15.8)	51	(16.4)
**Marital status**						
Single	47	(7.6)	20	(6.6)	27	(8.7)
Living with a spouse	329	(53.6)	217	(71.1)	112	(36.0)
Widowed/separated/divorced	240	(38.8)	68	(22.3)	172	(55.3)
**Type of family**						
Nuclear family	327	(53.1)	167	(54.8)	160	(51.4)
Joint or extended family	68	(11.0)	30	(9.8)	38	(12.3)
Three generation family	221	(35.9)	108	(35.4)	113	(36.3)
**Comorbidities**						
Absent/ unknown	111	(18.0)	64	(21.0)	47	(15.1)
1 disease	248	(40.3)	137	(44.9)	111	(35.7)
2 diseases	158	(25.6)	66	(21.6)	92	(29.6)
≥3 diseases	99	(16.1)	38	(12.5)	61	(19.6)

Table 2 describes the social characteristics of the participants by gender. Of the male participants, 44.3% stated that they had mutual interactions with their neighbors and 33.4% reported that they had no intimate friends. Of the female participants, 42.4% had mutual interactions with their neighbors and 39.9% had no intimate friends. Regarding religious habits, 89.2% of the males and 93.0% of the females performed praying/worshiping/meditation daily, while 69.8% of the males and 75.6% of the females donated food and other items to monks or poor people once a week or more. Most participants (88.2% of the males and 82.6% of the females) went outside their houses once a week or more. In total, 31.4% of the males and 12.6% of the females participated in community or social group activities at the time of the survey.

**Table 2:** Social characteristics of the study participants.

Variables	Total		Male		Female	
	(N = 616)		(N = 305)		(N = 311)	
	N	(%)	n	(%)	n	(%)
**Interaction with neighborhood**						
No interaction	14	(2.3)	10	(3.2)	4	(1.3)
Exchanging greetings	134	(21.8)	60	(19.7)	74	(23.8)
Daily chatting	201	(32.6)	100	(32.8)	101	(32.5)
Mutual consultation	267	(43.3)	135	(44.3)	132	(42.4)
**Having intimate friends**						
No friend	226	(36.7)	102	(33.4)	124	(39.9)
1–2 friends	143	(23.2)	62	(20.3)	81	(26.0)
3–5 friends	133	(21.6)	70	(23.0)	63	(20.3)
≥6 friends	114	(18.5)	71	(23.3)	43	(13.8)
**Praying/worshiping/meditation**						
Daily	562	(91.2)	272	(89.2)	290	(93.0)
Not daily	54	(8.8)	33	(10.8)	21	(7.0)
**Frequency of donation**						
Once a week or more	448	(72.7)	213	(69.8)	235	(75.6)
Less than once a week	168	(27.3)	92	(30.2)	76	(24.4)
**Frequency of going out**						
Once a week or more	526	(85.4)	269	(88.2)	257	(82.6)
Less than once a week	90	(14.6)	36	(11.8)	54	(17.4)
**Participation in group activities**						
Present	134	(21.9)	95	(31.4)	39	(12.6)
Absent	479	(78.1)	208	(68.6)	271	(87.4)

The WHOQOL-BREF scores for physical health, psychological health, social relationship, and environment domains and the distribution of domain scores according to age groups, gender, and residence by applying independent *t*-test are shown in Table 3. Of the four domains, the mean score for social relationship domain was 61.2 (SD 15.8), followed by psychological health 56.8 (SD 16.8), environment 54.8 (SD 15.6), and physical health 50.9 (SD 11.8). Participants aged 60–69 years had significantly higher QOL scores in the physical health domain. Male participants had significantly higher scores than females in the psychological health, social relationship, and environment domains. Participants who lived in urban areas of Yangon had higher QOL scores in all domains compared to those living in peri-urban areas based on respective p values.

**Table 3:** WHOQOL-BREF scores of study participants according to age, gender and residence (N = 616).

Characteristics	Number of participants	Physical health	Psychological health	Social relationship	Environment
	N	Mean (SD)	Mean (SD)	Mean (SD)	Mean (SD)
**Total**	616	50.9 (11.8)	56.8 (16.8)	61.2 (15.8)	54.8 (15.6)
**Age (years**)					
60–69	352	52.1 (11.2)	57.4 (17.0)	62.0 (16.2)	55.6 (15.6)
≥70	264	49.4 (12.4)	56.0 (16.5)	60.2 (15.2)	53.6 (15.5)
*t*-test		2.77***	1.01	1.41	1.58
**Gender**					
Male	305	51.8 (12.6)	58.4 (17.5)	63.4 (16.5)	56.6 (15.8)
Female	311	50.1 (10.9)	55.3 (16.0)	59.0 (14.8)	52.9 (15.1)
*t*-test		1.79	2.26*	3.51***	2.81***
**Residence**					
Urban	294	53.0 (11.3)	60.8 (16.2)	62.8 (15.4)	59.4 (15.5)
Peri-urban	322	48.9 (11.9)	53.2 (16.6)	59. 8 (16.1)	50.6 (14.5)
*t*-test		4.32***	5.67***	2.34**	7.29***

SD = standard deviation.

^*^ **p value < 0.05,**

^**^ **p value < 0.01,**

^***^ **p value < 0.001.**

Table 4 presents the results of the multiple linear regression analysis. Regarding the physical health domain, living in peri-urban areas, having no participation in group activities, and going out less than once a week were associated with a lower QOL for participants. Having low income compared to having no income, having ≥6 intimate friends compared to having no intimate friend, having more interaction with the neighborhood compared to having no interaction increased the QOL scores for the elderly in this study.

**Table 4:** Coefficients (β) and 95% Confidence Interval (CI) of predictors for WHOQOL-BREF of elderly people (N = 616).

	Beta coefficient (95% CI)			
Predictors	Physical health	Psychological health	Social relationship	Environment
**Residence**				
Urban	0 (Reference)	0 (Reference)	0 (Reference)	0 (Reference)
Peri-urban	-3.41 (-5.57, -1.25) **	-3.85 (-6.83, -0.87) *	-0.04 (-2.81, 2.73)	-6.00 (-8.66, -3.35) ***
**Education**				
High school level and above	0 (Reference)	0 (Reference)	0 (Reference)	0 (Reference)
No school/only read and write	0.79 (-2.19, 3.78)	-5.89 (-9.99, -1.77) **	-4.10 (-7.93, -0.28) *	-4.18 (-7.84, -0.51) *
Primary school level	-0.39 (-3.09, 2.30)	-5.53 (-9.25, -1.82) **	-3.09 (-6.55, 0.35)	-5.02 (-8.33, -1.71) **
Middle school level	-0.69 (-3.54, 2.14)	-3.66 (-7.57, 0.26)	-4.80 (-8.44, -1.16) *	-5.69 (-9.18, -2.19) **
**Marital status**				
Living with a spouse	0 (Reference)	0 (Reference)	0 (Reference)	0 (Reference)
Single	-0.94 (-4.48, 2.61)	-1.61 (-6.49, 3.28) *	-5.20 (-9.74, -0.66) *	-3.25 (-7.6, 1.10)
Widowed/separated/divorced	-1.95 (-4.12, 0.22)	-3.79 (-6.77, -0.80) *	-3.63 (-6.41, 0.86) *	-3.04 (-5.69, -0.38) *
**Income**				
No income	0 (Reference)	0 (Reference)	0 (Reference)	0 (Reference)
Low income	3.95 (1.80, 6.09) ***	4.25 (1.29, 7.21) **	2.87 (0.12, 5.62) *	5.17 (2.53, 7.81) ***
High income	1.59 (-1.04, 4.22)	3.22 (-0.40, 6.84)	-0.27 (-3.63, 3.09)	2.30 (-7.61, 1.10)
**Comorbidities**				
Absent/unknown	0 (Reference)	0 (Reference)	0 (Reference)	0 (Reference)
1 disease	-0.61 (-3.09, 1.87)	-2.98 (-6.41, 0.44)	2.87 (-0.31, 6.05)	-0.58 (-3.63, 2.47)
2 diseases	-2.50 (-5.20, 0.20)	-6.20 (-9.93, -2.48) **	2.48 (-0.98, 5.94)	-3.80 (-7.13, -0.48) *
≥3 diseases	-1.92 (-4.91, 1.12)	-10.53 (-14.73, -6.33) ***	3.00 (0.89, 6.91)	-5.89 (-9.64, -2.15) **
**Interaction with neighborhood**				
No interaction	0 (Reference)	0 (Reference)	0 (Reference)	0 (Reference)
Exchanging greetings	10.29 (4.15, 16.43) **	3.19 (-5.27, 11.66)	8.09 (0.23, 15.97) *	8.24 (0.69, 15.78) *
Daily chatting	10.77 (4.66, 16.88) **	2.44 (-5.98, 10.86)	9.27 (1.44, 17.09) *	8.47 (0.96, 15.98) *
Mutual consultation	11.51 (5.41, 17.61) ***	3.25 (-5.16, 11.66)	14.21 (6.39, 22.03) ***	10.53 (3.03, 18.03) **
**Having intimate friends**				
No friend	0 (Reference)	0 (Reference)	0 (Reference)	0 (Reference)
1–2 friends	0.89 (-1.50, 3.28)	1.77 (-1.53, 5.06)	3.15 (0.09, 6.22) *	1.42 (-1.52, 4.36)
3–5 friends	1.94 (-0.56, 4.44)	2.01 (-1.43, 5.45)	7.01 (3.81, 10.21) ***	3.47 (0.39, 6.54) *
≥6 friends	4.62 (1.95, 7.29) **	8.69 (5.01, 12.38) ***	12.89 (9.47, 16.32) ***	9.79 (6.51, 13.08) ***
**Praying/worshiping/meditation**				
Daily	0 (Reference)	0 (Reference)	0 (Reference)	0 (Reference)
Not daily	-1.36 (-4.66, 1.93)	-2.00 (-6.83, -0.87)	-6.52 (-10.73, -2.29) **	-2.24 (-6.29, 1.80)
**Frequency of going out**				
Once a week or more	0 (Reference)	0 (Reference)	0 (Reference)	0 (Reference)
Less than once a week	-2.79 (-5.34, -0.24) *	-2.41 (-5.92, 1.10)	-1.85 (-5.11, 1.42)	-2.65 (-5.78, 0.49)
**Participation in group activities**				
Present	0 (Reference)	0 (Reference)	0 (Reference)	0 (Reference)
Absent	-3.97 (-6.28, -1.67) **	-4.20 (-7.38, -1.03) *	-1.52 (-4.47, 1.44)	-3.18 (-6.02, -0.35) *

^*^ **p value < 0.05,**

^**^ **p value < 0.01,**

^***^ **p value < 0.001.**

Adjusted for age, gender, residence, education, religion, occupation, marital status, working status, income, comorbidities, type of family, interaction with the neighborhood, having intimate friends, praying/worshiping/meditation, frequency of donation, frequency of going out, and participation in group activities.

For the psychological health domain, living in peri-urban areas, having no formal education and having a primary school education level compared to having a high school education level and above, being single, being widowed/separated/divorced compared to living with a spouse, having two or more co-morbidities, and having no participation in group activities were significantly associated with lower QOL scores for the participants. Having a low income compared to having no income and having ≥6 intimate friends compared to having no intimate friend were associated with higher QOL scores in the psychological health domain.

Concerning the social relationship domain, having more interaction with neighbors, having one or more intimate friends, and having low income compared to having no income were significantly associated with higher QOL scores for the participants. Having no formal education or having a middle school education level compared to having a high school level and above, being single and being widowed/separated/divorced compared to living with a spouse, and not praying/worshiping/meditation daily significantly reduced the QOL scores in this domain.

Regarding the environment domain, having three or more friends, having more interaction with neighbors, having low income compared to having no income were associated with higher QOL scores. Meanwhile, having no participation in group activities, living in peri-urban areas, having lower education than a high school level, being widowed/separated/divorced, and having two or more co-morbidities were associated with lower QOL scores.

## Discussion

This is the first study reporting on factors associated with QOL for elderly in the Yangon Region, Myanmar. Income level and having intimate friends influenced on the QOL scores in all domains, meantime, education level and marital status influenced on psychological health, social relationship and environment domains. Social interaction with neighbors increased the QOL scores for the physical health, social relationship, and environment domains. Living in peri-urban areas was associated with lower QOL scores for the physical health, psychological health, and environment domains, while participation in group activities increased QOL scores in these domains. Having comorbidities affected the QOL in the psychological health and environment domains, while the frequency of going out affected physical health and the frequency of religious practices impacted social relationship.

Compared to the elderly with no income, those with low income reported having a higher QOL in all four of the domains; physical health, psychological health, social relationship, and environment domains. This aligns with previous studies that also reported income was significantly associated with QOL in the elderly [15, 16, 19]. Even having low income from at least one source is better than having no income at all. Myanmar Aging Survey in 2014 reported that 60% of the elderly lived in households with incomes of no more than three USD per day [14, 20]. Poverty may result in limited access to basic services and needs for the elderly in health care, transportation, education, and locating safe and age-friendly living places [21]. This could affect the QOL for the elderly in the physical health, psychological health, social relationship, and environment domains. Moreover, the social security system and support for the elderly population are still insufficient in Myanmar, although the new government has started introducing new social security programs for the elderly. It made the elderly participants to rely mainly on their own income and financial support from their families and relatives. All of these factors could be the reasons why elderly participants with a low income had a higher QOL than those with no income.

There was no significant difference in the QOL for the four domains between elderly with a high income and elderly with no income. In the current study, only 16.1% of participants had a high income, while the majority of the participants had either low or no income. Among the low-income group, more than one-third were currently working. However, in the high-income group, only one in four were currently working, which suggests that the higher income may be from the financial support of their family or from their pension. Similar to other studies, our findings showed that employed elderly individuals had a higher QOL than unemployed [7, 22]. Therefore, employment status may have an indirect effect on QOL for the low-income group, as there was a significant association between low-income and QOL scores for the low-income, but not the high-income group.

In this study, the elderly living in peri-urban areas had lower QOL scores for the physical health, psychological health, and environment domains. This contradicts findings reported in a study from Thailand where Thai people living in suburban areas had a higher QOL than those living in urban areas [23]. This may be due to differences in demographics, study populations, and economic development. The urban poverty rate in Yangon was 34.6% in 2014 and there were expansions in the area known as the “slum”, which reflected the poorer economic and environmental conditions in the peri-urban area [24]. As the economic growth and the infrastructure of the urban and peri-urban areas of the Yangon Region are different, there may be differences in healthcare, education, job opportunities, safe drinking water, waste disposal, and convenient public transportation, which could lead to lower QOL in the physical health, psychological health, and environment domains. The QOL scores for the social relationship domain among the elderly from urban and peri-urban areas showed no significant difference because it is likely that the elderly population from the Yangon Region share similar social values and customs regardless of where they are living. To improve the QOL for the elderly living in peri-urban areas, economic development and financial assistance programs for healthcare should be encouraged in the Yangon Region.

In the current study, elderly with ≥6 intimate friends reported to have higher QOL scores in all four domains compared to those with no intimate friend. In addition, elderly individuals with more interactions with neighbors had higher QOL scores for the physical health, social relationship, and environment domains compared to those with no interaction with neighbors. The elderly who engaged in group activities had good QOL scores for the physical health, psychological health, and environment domains. Older people are vulnerable to loneliness and social isolation [25]. Many researchers have pointed out that social roles and participation in group activities are important factors and recommended for promoting mental and physical well-being and QOL for elderly people [19, 25, 26]. Retiring from their full-time job and fewer responsibilities within the family may create more free time for the elderly to interact with their friends or neighbors. The Myanmar Aging Survey in 2014 reported that nearly two-thirds of the elderly socialized with friends or neighbors [14]. By engaging in social interactions with friends and neighbors, and by participating in group activities, the elderly can gain access to new information, make new friends, and have opportunities to participate in community activities for an active physical and social life. Thus, the families, relatives, friends, and community of the elderly should encourage more social interaction and participation for better physical, mental, social well-being, and QOL. Special attention should be given to persuade and support the elderly with no social interaction and no social participation to enjoy interactions with neighbors, friends, and communities.

Even though the current study findings indicate that the elderly have good QOL because of more intimate friends, good interactions with neighbors, and participation in group activities. However, this kind of social relationship could be bi-directional because the elderly people who had good QOL may be more motivated and active to be involved in social interaction and participation. Due to the cross-sectional study design, the causal relationship between these variables and QOL may not be precisely determined.

Compared to the elderly who were currently living with their spouses, those who were widowed, separated, or divorced had lower QOL scores for the psychological health, social relationship, and environment domains and those who were single reported having lower QOL scores for the psychological health and social relationship domains. Regarding the marital status of the elderly, studies have reported that overall well-being and QOL were lower in single elderly (unmarried and widowed), and that family relationships are critical for the elderly in general [27, 28]. Moreover, their spouses may play the role of primary provider for material, financial, social, emotional support, and personal care when getting older or during illness [14]. However, the elderly cannot deny the decline in physical conditions and functional limitation due to aging, no matter how much support and care they receive from their spouses. This could explain why the physical health QOL score was not influenced by marital status.

The current study findings also showed that elderly with lower education had lower QOL scores for the psychological health, social relationship, and environment domains, compared to those with a high school education level and above. Studies from other countries have also reported that education is one of the best predictors of longevity and influenced on QOL of the elderly [12, 29–31]. Better education may create better job opportunities, better income, and better living standards, which in turn result in higher QOL scores for the psychological health and environment domains. In addition, an educated person has the ability to decide independently. Education challenges people to learn about essential parts of their surroundings and values in their society, helping people to build meaningful external ties with the community, which may result in better social relationships.

More than 80% of the elderly in this study reported having at least one disease, mostly non-communicable diseases. Elderly participants with more comorbidities were reported to have lower QOL scores in the psychological health and environment domains. Associations between chronic diseases and lower QOL in the elderly have been mentioned in various studies [32–34]. A possible explanation for lower scores in the psychological health domain may be due to worrying about the diseases, complications, medical expenses, and need for assistance with daily activities. Myanmar is a low-middle income country in Southeast Asia with limited resources. Considering the chronic nature of non-communicable diseases and the rise in the elderly population, Myanmar should strengthen its existing elderly healthcare services and social support systems to ensure better QOL in the elderly population.

Elderly individuals who performed daily praying/worshiping/meditation had higher QOL scores in the social relationship domain. The elderly Myanmar people usually perform praying/worshiping/meditation daily at home or at the nearby pagodas, temples, and monasteries. Visits to monasteries, pagodas, and temples enable them to socialize with other people, share information with each other, and enjoy their leisure time, which could have a positive effect on social relationships. One systematic review showed that religiosity helped older people in dealing with their losses and their difficulties. The majority of the analyzed studies (75% of the studies in that systematic review) showed a positive association between religious involvement and QOL for older adults in mental, social, and physical aspects [35].

This study has several limitations. First, there was a possibility of recall bias in the participants’ reported answers because some questions required detailed memories of past events. Second, underreporting of comorbidities might have affected the study findings because only diagnosed diseases were considered. Third, the findings from this study might not be generalizable to the whole elderly population in Myanmar because it was conducted in only four townships in the Yangon Region. Fourth, considering the subjective nature of QOL and the influence of multiple unique factors encountered throughout life, assessing QOL with the variables used in this study may not be sufficient for capturing all aspects of the elderly perspective. Further, in-depth studies on the QOL of the elderly population in Myanmar are recommended for a better understanding of QOL. Finally, due to the cross-sectional study design, the causal relationship between the independent variables and the QOL for the elderly could not be confirmed, and there may be possibilities of the reverse effects between them.

## Conclusion

In conclusion, residential location, education level, marital status, income, comorbidities, social interactions with neighbors and friends, participation in group activities, and frequencies of going out and religious activities in the elderly were factors that should be considered in planning and implementing programs for the elderly in Myanmar. Peri-urban development, strengthening healthcare and social security systems, and encouraging social interaction and participation in group activities play critical roles in improving the QOL for the elderly residing in the Yangon Region.
